# Discussion

**Published:** 1918-10

**Authors:** 


**Discussion.**

MAJOR ZINSSER was asked by Colonel Gressinger to state the results of the bacteriological examination of the feces in the cases of diarrhea which he had studied. He said that the examination was not complete but it showed very definitely that the disease was of a bacillary nature. There were thousands of cases, and only the severe ones were picked out and sent to a hospital where they were studied clinically and the early stools examined. The methods used were the simple methods used for army work. The result of one series of examinations showed that out of 35 cases, 2 were paratyphoid. A great many cases showed a curious organism which he hoped to study more carefully because he had never seen it before; it did not correspond to any of the ordinary types one sees in feces, though it was like dysentery or typhoid organisms. He said that he had left instructions to do about 300 more stools. There was some question of the diarrhea coming from infected food, but he did not know what was found subsequently. He thought, however, that with conditions such as those just described every kind of epidemic might be expected, and he suggested protecting the men by vaccination whenever possible.

Captain Moss asked to be excused from discussing the subject of fighting flies and disposing of feces in France. He said he should feel very badly to think that epidemic diseases were spreading into advanced areas, and very humiliated to think that they started in base sections. He believed there was no necessity of discussing general principles about flies and feces, nor the matter of expediency. There was little to say about this problem at a base section that would be of interest. Various methods which seemed to be satisfactory had been tried, but conditions were very different from those in the front area.

Captain Moss said he should like, however, to say a word about dysentery. There had been reports of dysentery from a number of localities in his base section. Diarrhea had occurred in some places in considerable amounts; it had not been found possible to go out and make a diagnosis in every case, but each locality where diarrhea was prevalent had been visited to determine whether the outbreak was dysentery or not. In every instance a sufficient number of specimens had been obtained, and dysentery bacilli isolated from the stools. This had not occurred in every case, but out of 20 specimens of dysentery, 5 or 10 cases gave evidence that it was true dysentery.

Observations in these cases led to the belief that the epidemics were fly-borne. Water throughout the base sections was being conscientiously chlorinated and there was no reason to believe that the dysentery was anything but fly-borne.

Any success that he and his colleagues had had in dealing with epidemics of this kind had been very largely due to the splendid co-operation of the Commanding General. He had been willing to take circulars or bulletins, which had been prepared, and put them out under the form of general orders and over his signature. We had tried to carry out the method of anticipation, — preparation, co-operation, and investigation — to make provision a little in advance, and to get out circulars on diseases for which the medical officer was on the watch. An attempt had been made to get ready to supply any demands which the appearance of those diseases might cause the medical officer to make upon the base regions. If the diseases did occur, there would be co-operation with the medical officer in the diagnosis. This had already been done in the case of dysentery, meningitis, pneumonia, etc.

Base sections in other parts of France had received warning from circulars which had preceded the diseases, and the speaker believed that if some educational policy could be carried out before the winter came on, a great deal in preventive medicine would be accomplished.

The speaker asked Colonel Siler to give his views.

Colonel Siler said that his office was now getting reports from the base hospitals through which the men were evacuated. Two days previously 5 cases of typhoid and 12 cases of dysentery had been reported. The latter had not yet been confirmed bacteriologically, but they were doubtless cases of dysentery. Careful investigation showed absolutely no dysentery of previous attacks; they were all primary cases. Colonel Siler also said that a number of cases of dysentery had been proved to be due to the entamoeba hystoliticus, and suggested that there might be many more cases of that type.

Médecin-Major Rist said he could confirm what Colonel Siler said. The French physicians had been surprised to find that a great number of cases, which at the beginning were thought to be bacillus cases, were in reality primary dysentery.

Colonel Cummins said that the British medical officers had found an organism which was probably.the one mentioned by Major Zinsser. This organism can be definitely set aside on account of its strange fishy odor.

This year it had been arranged that cases of diarrhea occurring in a division should be brought to the field ambulance at once. The command does not want to lose the men from the division; therefore, instead of sending them to the clearing station, those who are comparatively slight cases are kept in the field ambulance. Swabs are taken and sent to the bacteriologist by a motor cyclist. One motor cyclist can carry about 100 dysentery swabs at a time. The bacteriologist deals with these swabs at once and generally submits his report within 4 or 6 hours. This system has enabled the authorities more and more to find the dysentery cases among the diarrhea cases, but not to a complete extent, because in the case of diarrhea dysentery as in the case of clinical dysentery the men do not report. In one army, from the divisisions that followed instructions, there was as much as 23 o/o of dysentery positive findings from the cases of diarrhea examined.

Instructions had been given to get all cases of diarrhea brought in and observed. As a result a considerable number of diarrhea cases had been removed from the line and it had been found necessary to send a lot of them back to the base because solid food brought on looseness of the bowels very quickly again. If these cases had not been treated they would have relapsed into dysentery and become chronic.

If all cases of diarrhea can be examined and treated this year, the number will probably be diminished next year.

At the present moment there are a number of cases of dysentery or suspected dysentery. Evacuation to England is prevented because it is considered that these men will soon get well if properly treated.

Many cases of minor illness are kept back, which last year would have been evacuated to England and the majority of them can be cured within one month.

In regard to the question of sanitation, which is so important, the speaker said that it was a very good thing to say " Stop the flies, destroy the flies ". The speaker advocated fly destruction in every way, but we must bear in mind the principle that the fly is merely an instrument in the transmission of infected matter to food. The importance of flies is enormous, but the importance of feces is far greater. So many agents besides flies are important, for instance, the men's boots. When walking over a bad system of latrine a man will come back with his boots foul; he will lie down among other men and rub his boots on them, or he will take his boots off, thereby bringing his hands in contact with them.

The covering up of food to protect it from flies is very important, but the great thing is to remember what food ought to be covered up. It is a very good thing to see a screened meat-safe, but the joints of meat that are in it will be cooked, while outside the screen you may see cheese, sugar, etc., which are not to be cooked. Food that is not to be cooked should be covered. One is prescribing means and not ends.

If a kitchen has good sanitation, but in the mess-room jam is uncovered, sugar exposed, and bread uncovered, the good sanitation of the cook is lost to a certain extent. The authorities should think of what they are aiming at rather than concentrate on one particular example.

